# Methicillin-resistant *Staphylococcus aureus* prevalence in food-producing animals and food products in Saudi Arabia: A review

**DOI:** 10.14202/vetworld.2024.1753-1764

**Published:** 2024-08-13

**Authors:** Dalal M. Alkuraythi, Manal M. Alkhulaifi

**Affiliations:** 1 Department of Biology, College of Science, University of Jeddah, Jeddah, Saudi Arabia; 2 Department of Botany and Microbiology, College of Science, King Saud University, Riyadh 11451, Saudi Arabia

**Keywords:** antibiotic resistance, food-producing animals, livestock-associated methicillin-resistant *Staphylococcus aureus*, methicillin-resistant *Staphylococcus aureus*, Saudi Arabia

## Abstract

In Saudi Arabia, the occurrence of methicillin-resistant *Staphylococcus aureus* (MRSA) in food and livestock represents a major public health hazard. The emergence of livestock-associated MRSA has heightened the risk of human infection with comparable virulence traits. The lack of information about MRSA transmission in our region hinders accurate risk assessment, despite its detection in food animals and retail foods. Adopting a One Health approach is essential for effectively combating MRSA in Saudi Arabia. This method unites actions in the human, animal, and environmental spheres. To combat MRSA contamination, surveillance measures need strengthening; interdisciplinary collaboration among healthcare professionals, veterinarians, and environmental scientists is crucial, and targeted interventions must be implemented in local food chains and animal populations. Through a holistic strategy, public health and sustainable food production in the region are protected. This review aims to improve public health interventions by increasing understanding of MRSA prevalence and related risks in local food chains and animal populations.

## Introduction

*Staphylococcus aureus*, occurring naturally in humans and animals, can inhabit the skin and mucous membranes such as the respiratory, gastrointestinal, and genitourinary tracts [1]. Nonetheless, *S. aureus*, an opportunistic bacterium and one of the most significant bacterial pathogens [2], is carried by roughly one-third of the population in their noses, with about 2%–3% of these individuals carrying methicillin-resistant *S. aureus* (MRSA), and this figure rises to approximately 5% for healthcare providers [3]. The prevalence of MRSA among *S. aureus* strains in Saudi Arabia was estimated to be 35.6% as determined by a pooled analysis of over 22,000 *S. aureus* strains [4].

The discovery of MRSA in domestic and livestock animals has raised interest in the genetic development of *S. aureus* as a zoonotic foodborne pathogen [5]. Recently, the extensive use of antibacterial drugs has had a significant impact on the evolution of *S. aureus* in both animals and humans, as evidenced by the rise of livestock-associated MRSA (LA-MRSA) strains [5]. MRSA transmission from food-producing animals to humans was first discovered in 2005 in swine, when pig farmers and their families were found to be either carriers or infected with LA-MRSA sequence type (ST398) [6]. This new type of isolate belonging to clonal complex 398 (CC398) comprises the main MRSA reservoir outside hospitals [7]. Initially, this MRSA isolate was found to exist among pigs in Europe and soon after it colonized many species of food-producing and companion animals [7]. LA-MRSA transmission occurs primarily in humans interacting with livestock and secondarily through contaminated meat products [8]. Other MRSA variants, such as MRSA-infected retail meat, can also be transmission sources [9]. Raw meat can easily be contaminated during butchering, handling, transportation, and storage through contact with blades, utensils, clothes, and hands [10]. Consumers and others who handle raw meat may come in contact with MRSA through contaminated meat; these strains may cause infections such as *S. aureus* or MRSA in humans [11]. Retail meat from animal sources plays an essential role in the development of antimicrobial resistance [9]. MRSA has been isolated from various meat and poultry products, and its transmission between food-producing animals and humans has been established [12]. Notably, the risk of MRSA infection differs from that of *S. aureus* food poisoning, which is a prevalent food safety issue [13]. The latter occurs when some *S. aureus* strains capable of producing toxins contaminate and grow in food under toxin-producing conditions [13]. Clinical signs of *S. aureus* food poisoning vary depending on the toxin and dosage consumed but are typically minor gastrointestinal symptoms that spontaneously recover without the need for a particular treatment [14]. In contrast, invasive MRSA may cause skin and wound infections as well as more serious infections, including pneumonia and bloodstream infections that lead to sepsis [2]. Since community-associated MRSA (CA-MRSA) is said to have less serious implications than hospital-acquired MRSA (HA-MRSA), Saudi researchers are concentrating more on the latter in their studies than LA-MRSA and other types of MRSA [15]. Studying MRSA in humans, food, and animals in terms of dissemination, antibiotic resistance, and clonal lineages is of substantial relevance because Saudi Arabia is one of the most populous and largest countries in the Middle East [4, 16]. Human movements (e.g., migration, tourism, and pilgrimages) in Saudi Arabia are primarily responsible for the dissemination of bacterial species and their clones [17]. Therefore, Saudi Arabia is frequently a hotspot for the prevalence of novel MRSA clones with newly emerging genotypes and phenotypes.

This review examines the frequency, dissemination, and consequences of MRSA in Saudi Arabia, emphasizing its occurrence in humans, animals, and the food industry. The article will delve into the emergence of livestock-associated MRSA strains and the role of extensive antibacterial drug use in shaping their evolution. The review synthesizes recent research findings to improve knowledge of MRSA dynamics in Saudi Arabia, guiding control and prevention efforts.

### MRSA occurrence in humans, animals, and food

At least three distinct settings, that is, hospitals, communities, and farms, have given rise to MRSA clones that have occurred at different times and locations [13]. Most MRSA strains were identified as HA-MRSA when they were initially discovered in the 1960s because they were associated with nosocomial infections in healthcare facilities [18]. However, in the early 1990s, individuals living in rural communities with no history of hospitalization were found to be infected with CA-MRSA [19]. Thereafter, new MRSA strains that were first reported in livestock in 2005 and designated LA-MRSA emerged [19]. Because of the continued spread of these MRSA clones over time, certain hospital isolates are now present in the community and vice versa, and human cases of livestock strains are also increasing [20].

### MRSA

Through staphylococcal cassette chromosome mec (SCC*mec*) molecular typing and phylogenetic analysis, HA-MRSA and other MRSA types are accurately defined [2]. SCC*mec* is a mobile genetic element carrying the *mecA* gene and is responsible for methicillin resistance [21]. SCC*mec* types are classified based on two main components: The *mec* and *ccr* gene complexes [21]. Resistance to *β*-lactams is conveyed by SCC*mec* types I to III in HA-MRSA, whereas resistance to *β*-lactam antibiotics is conveyed by SCC*mec* types IV and V in CA-MRSA [22]. SCC*mec* IVa and V are the most prevalent SCC*mec* cassettes found in LA-MRSA, whereas SCC*mec* type XI, which contains *mec*C, has been identified in this MRSA strain [23].

HA-MRSA is the most common cause of hospital-based infections [24]. Although it was discovered in 1961, it did not pose a serious threat until the mid-1980s [24]. MRSA infections in hospitalized patients are associated with age, prolonged hospital stay, catheter use, and equipment use [25]. HA-MRSA is the dominant MRSA clone that causes most MRSA infections, in which successful clones of HA-MRSA tend to be transferred between hospitalized patients [26]. The distribution of thriving HA-MRSA clones varies geographically; in the US, the most dominant clone is USA100 (also known as the CC5 clone), which was recently surpassed by the CA-MRSA USA300 (also known as CC8 clone) [27]. The clonal complexes CC8 and CC5 also account for the majority of HA-MRSA clones found in South America [28]. The most common European isolates of HA-MRSA include CC5, CC45, CC22, and CC8 [29]. CC8/ST239 is a major hospital-acquired clone in African countries [30–33]. The majority of circulating HA-MRSA clones in Australia and New Zealand were CC8 [34, 35].

CA-MRSA has recently experienced clinically significant outbreaks linked to severe infections, particularly among those who are not hospitalized [2]. CA-MRSA was the first confirmed case in the 1990s in Western Australia, despite initial reports in the 1980s in Detroit and Dublin [36–38]. Since CA-MRSA does not possess non-β-lactam antibiotic resistance genes due to its absence in CA-MRSA’s gene composition, new CA-MRSA clones are believed to originate from the incorporation of SCCmec into MSSA strains [39]. CA-MRSA is now almost entirely responsible for endemic hospital-acquired infections, replacing HA-MRSA clones [40]. Approximately 20 distinct genetic lineages have been attributed to CA-MRSA [41]. The most common CA-MRSA clones worldwide include CC30-IV, CC15-IV, CC59-V, and CC80-IV, whereas USA300 is most common in North America (CC8) [41–44]. Because ST8-IV (CC8) and ST30-IV (CC30) are continuously isolated from all continents, they can be regarded as pandemic CA-MRSA clones [45].

Since its discovery in Belgium in 1972, MRSA epidemiology has undergone significant changes, raising concerns about its prevalence in animals [46]. LA-MRSA was first reported in pigs and pig farmers in 2005 [47]. The LA-MRSA isolate, genetically distinct from human MRSA isolates [13], represents the primary MRSA reservoir outside hospitals. This clone has been associated with meat-producing animals, such as pigs and calves, although it can colonize and cause infections in several hosts, including sheep, goats, chickens, rabbits, horses, and companion animals, such as dogs and cats [48–50]. From an economic perspective, LA-MRSA strains are important because they infect and cause economically important diseases in farm livestock [51].

### *S. aureus* as a pathogen adapted to animals

Although some bacteria can infect several hosts, many pathogenic bacteria are host-specialized and uniquely adapt to and evolve within a single host [52]. Some strains of *S. aureus* have a substantial ability to colonize and infect various hosts in addition to humans [1]. Some MRSA strains that originated in humans have moved to various animal hosts, where they have developed and adapted by disposing of virulence characteristics that are useless in the new host and obtaining new characteristics that improve bacterial fitness in the new environment [13]. The first instance of MRSA transmission between humans and animals was documented more than 20 years ago, raising the question of whether healthy animals can serve as reservoirs [53]. CC398 was first identified as a “swine” clone and has since been found in humans and other animals [6]. Other MRSA clones that are largely associated with human infections, such as MRSA ST1, ST5, and ST130, have also been found to infect animals. Although it is a common cause of infection in humans, it has also been linked to animal infections, such as bovine mastitis in cows [54]. Interspecies transmission and host jumps are of utmost importance because some of the most destructive disease epidemics have been caused by switching from one host species to another, including influenza, acquired immune deficiency syndrome, plague, and smallpox [55]. Through genetically defined mechanisms that cause it to have a specialized affinity for particular bacterial receptors to proteins of the preferred host, it adapts particularly to specific animal hosts. However, because of the plasticity of its genome, MRSA is a pathogen notorious for its high jump rate [1]. Over the years, new MRSA clones have emerged from humans and animals [56]. Significant host-switching events have also been reported. For example, the sequence types of *Staphylococcus argenteus* are more closely related to *the S. aureus* sequence types of bat and monkey isolates than to other human *S. aureus* sequence types, indicating their non-human origin [57].

### MRSA in animal food

MRSA can be found on various meat and poultry products and transfer through contaminated food [13]. MRSA infection risk differs significantly from *S. aureus* food poisoning, acknowledged as a serious food safety concern [13]. Food contamination and toxin production by certain *S. aureus* strains occur under suitable conditions (temperature and food environment) [13]. Clinical symptoms of *S. aureus* food poisoning are often mild gastrointestinal symptoms that resolve within 24–48 h without the need for medical attendance [58]. However, these symptoms may vary depending on the toxin and dosage consumed [58]. LA-MRSA ST398 is mainly found in foods of animal origin meant for human consumption, especially meat, although CA-MRSA and HA-MRSA may also be detected, indicating that humans are a potential source of contamination, possibly during slaughter or processing [59].

### Dissemination of MRSA in food and animals and its significance for health

**Figure-1:**
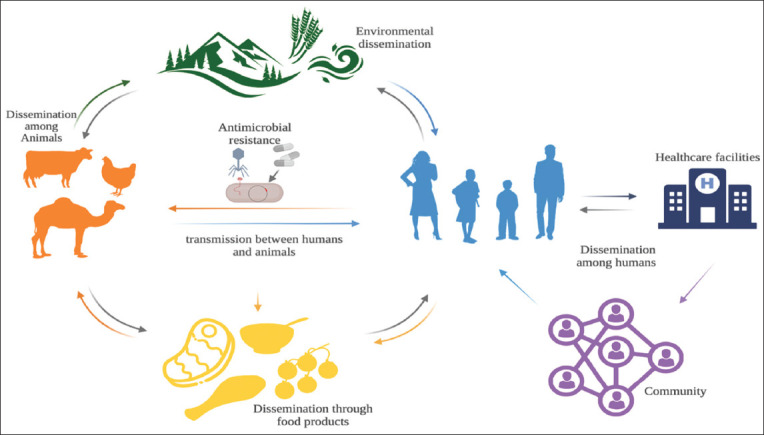
Conceptual schematic model of pathways of exposure and risks of methicillin-resistant *Staphylococcus aureus* transmission and dissemination between food, animals, and humans [Source: The figure was prepared using BioRender.com].

The presence of MRSA in various types of food, especially those of animal origin, has been demonstrated in many recent studies [11, 60–62]. Although the production of staphylococcal enterotoxins by *S. aureus* in food is a major cause of food poisoning, its presence in food does not pose a high risk to human health [63]. MRSA’s presence in food and animals poses a risk due to its ability to transmit between hosts and environments while evolving antimicrobial resistance. In slaughterhouses and meat shops, the presence of MRSA in animal carcasses can transfer to workers’ hands, surfaces, and tools; however, previous reports by Sadiq *et al*. [64] indicate a low risk of infection in such an environment. However, the rate of MRSA presence in meat-producing animals, farm workers, and their families is higher, and the risk of MRSA colonization and subsequent infection is feasible [65]. Due to the overuse of antibiotics by some farms to promote animal growth and protect them from diseases, MRSA in these animals may become resistant to antimicrobials, posing a new risk to public health [66]. The presence of MRSA in animals in close proximity to humans may be the most dangerous to public health, as studies have shown that many MRSA strains that infect companion animals are capable of causing infections in humans, similar to those usually found in hospitals [8]. These MRSA strains may act as vehicles to infect both humans and other animal species, allowing them to evolve within the host and acquire new traits that increase their virulence, such as antibiotic resistance [13] (Figure-1).

## Common MRSA Lineages in Animals and Humans

Although the major clones that cause CA-MRSA infections vary worldwide, there are some dominant clones (ST80, ST30, and ST8), whereas in Africa and Asia, no dominant clone has been detected [67]. In contrast, the most common HA-MRSA clones that cause infections are (ST5, ST239, and ST36) [68]. LA-MRSA, an important zoonotic pathogen, has also been identified in patients [20]. Among the LA-MRSA clones that have emerged in hospitals and caused human infections are CC398, CC97, and CC96 [69, 70]. Nonetheless, MRSA infection and colonization in companions and food-producing animals have been documented [20]. LA-MRSA sequence type ST398 was detected in meat products in China and the Netherlands [62, 71]. Although ST398 was the most prevalent sequence type in turkeys in the United States, ST5, ST1159, and ST1 were more frequent in chicken, beef, and pork, respectively, the presence of distinct *S. aureus* populations on each type of meat indicates that animals are the primary source of contamination [72]. In Georgia, none of the retail meat MRSA isolates were identified as ST398, despite the presence of other sequence types (STs) (ST5, ST8, ST9, and ST30), which have been recognized in some reports as LA-MRSA clones [70, 73]. ST5 and ST8 are human-associated MRSA types that are frequent in HA-MRSA and CA-MRSA, respectively, indicating that these meat products could be contaminated with isolates from human sources [73]. The major clones present in companion animals are ST22, ST5, and ST8, whereas the most common clones present in food-producing animals are ST398, ST5, ST 130, and ST9 [20]. Although the ST398 clone is mainly found in pigs and other animals, it also colonizes humans and causes infections similar to those of MRSA belonging to HA-MRSA and CA-MRSA [74].

## MRSA Association with Animals and Food Products in Middle Eastern Countries

Arab nations are mainly composed of Arab League’s 22 Arabic-speaking countries, which have a combined population of around 453 million people [75]. Arab countries are comprised of twelve Middle Eastern countries (Saudi Arabia, the United Arab Emirates, Bahrain, Iraq, Jordan, Kuwait, Lebanon, Oman, Palestine, Qatar, Syria, and Yemen) and ten North African countries (Egypt, Morocco, Algeria, Libya, Sudan, Tunisia, Mauritania, Djibouti, Comoros and, Somalia) [75]. However, there are insufficient reports on MRSA in animals and food in a few Arab countries [76]. In animals producing food, specifically cattle, the prevalence of MRSA in milk samples is between 3.60% and 35.7%, whereas 15.5%–40% of cattle nasals are reservoirs of MRSA [77]. The variation in MRSA prevalence between regions is mostly due to geographical diversity and variations in animal and food sources across countries [78]. A contributing factor to the increasing prevalence of MRSA in cattle may be the expanding mass production in cow rearing [79]. MRSA from lineages CC22, CC1, CC5, and CC45 has been detected in cattle in Egypt, although CC22, CC1, and CC5 are classified as HA-MRSA and have previously been prevalent in humans [80, 81]. In Arab countries, MRSA nasal carriage in sheep varied between 3% and 28.9%, and the highest frequency of MRSA was found in goats at 17.4% in milk samples, whereas only 2% was found in nasal swabs [77]. This variation in the prevalence of MRSA in sheep may be due to differences in sample sizes, sample collection methods, farm worker skills, and geographical locations [82]. A study in Algeria found that camels have MRSA because these isolates belong to the CA-MRSA CC80 (ST80 and ST152), which is one of the most reported CA-MRSA isolates in the Arab world [83]. Successful MRSA clones can adapt and spread owing to several factors, including the acquisition of antibiotic resistance genes, which enables them to rapidly adapt to antibiotic exposure and new hosts [84]. MRSA has been found in poultry in several Arab countries, where a long-term study from Algeria revealed an MRSA prevalence of 24.2%, whereas the frequency was comparable in Iraq and Egypt [85–87]. MRSA has been detected in various types of food in Arab countries, including Algeria, Tunisia, and Jordan, with prevalence rates of 18%–20% and 26%, respectively [88–90]. Unusually, raw meat in Tunisia was found to contain LA-MRSA ST398 [91].

## MRSA Epidemiology in Saudi Arabia

**Figure-2:**
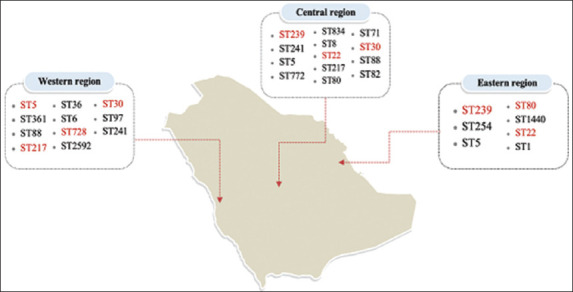
STs distribution of methicillin-resistant *Staphylococcus aureus* isolates from Saudi Arabia. Red colored STs represents the most common STs in the region.

Saudi Arabia is one of the most populated and largest countries in the Middle East, with an estimated population of 34 million [92]. Mass human movements (e.g., migration, tourism, and pilgrimages) contribute to widespread emerging strains of pathogens and their associated diseases [93]. Muslims from many countries regularly visit Saudi Arabia’s holy sites, which corresponds to the spread of bacterial species and their clones [17]. These conditions make Saudi Arabia a constant hotspot for the collection of new MRSA isolates with an emerging genotype and, consequently, phenotypes such as antibiotic resistance [17]. Successful MRSA clones are responsible for the rapid growth of the MRSA population in many parts of the world, and new MRSA strains have continued to develop and diminish for decades due to unknown factors [94]. Despite its global reach, what was once restricted to hospitals and healthcare settings has increasingly infiltrated society and has even arisen in livestock [94]. In Saudi Arabia, the distribution of MRSA clones in different regions varies [4, 17, 95, 96] (Figure-2). The prevalence of MRSA differs from region to region, and according to the findings of a 2018 meta-analysis, the prevalence of MRSA in Saudi Arabia is >35%, which is a considerable increase from the previously reported 29.9% by Adam and Abomughaid [97]. The analysis focused on two major regions of Saudi Arabia, the western and central regions, with prevalence rates of 42% and 32%, respectively. In cattle nasal swabs, there is a prevalence of 15.5% MRSA, whereas in goats, this percentage ranges from 2% to 28.9% [98, 99]. The most common circulating clones of CA-MRSA reported in Saudi Arabia are CC80-IV, CC22-IV, and ST30-IV, whereas there are some sporadic clones, such as CC6-IV and CC88-IV [95, 100]. In Saudi Arabia’s capital hospitals, cases have been recorded for the pediatric clone (CC5) as of 2015, in addition to the common CC8-ST239-III clone and other prevalent clones in Riyadh hospitals, including CC22-IV, ST30-IV, and the European CA-MRSA clone CC80-IV. CC8/ST239-III was previously found to be the most prevalent HA-MRSA epidemic clone in Saudi Arabia [4]. In the eastern region, a study found that the CC80 (ST80, ST1440) clone was the most often identified strain, followed by CC22 (ST22) [95]. In other studies, CC1, CC88, and CC80 102 were the predominant MRSA clonal lineages in the eastern region [96]. In the western region of Saudi Arabia, a study with diverse MRSA lineages including CC5, CC22, CC80, and CC30 was performed in 2019 by Al-Zahrani *et al*. [17]. The pandemic strain CC8-ST239-III, which was formerly widespread in the GCC, has drastically decreased since the emergence of CA-MRSA, but is still present in infrequent reports [101]. According to Senok *et al.*, only nine of 117 isolates were ST239 MRSA-III, whereas the remaining 117 strains were all CA-MRSA lineages because they carried SCC*mec* IV and V and belonged to 14 distinct clones of all the studied HA-MRSA strains, with the most prevalent being CC80, CC6, CC5, and CC22 [102]. Some known HA- and CA-MRSA clones, such as ST6-IV, ST5-VI, and ST5-V, are also prevalent in livestock and animals [103]. One unique clone associated with livestock that has been identified is CC15-ST1535-V, which comprises the staphylococcal cassette chromosome fus (SCC*fus*) composite element and is related to retail camel meat products in Saudi Arabia [104]. Another noteworthy new variant in Saudi Arabia is the PVL-producing CC1153-I+SCC*fus* unique clone, which contains another unusual composite element, SCC*mec* type V, with SCC*fus* [19]. All of the previously identified circulating MRSA clones that cause infections in Saudi Arabian hospitals and communities have also been found in animals, specifically in companion animals and livestock [20, 105, 106]. The prevalence of MRSA clones varies significantly within Saudi Arabia and the rest of the Middle Eastern countries because of several factors [107]. Regional factors (agricultural workers, weather, and land), infection prevention strategies, and transmission due to travel may have an impact on variations in MRSA clones in the Middle Eastern region [4]. The Western region of Saudi Arabia has the highest rate of MRSA prevalence, possibly because of the presence of holy Islamic sites that receive millions of pilgrims each year [96].

### MRSA prevalence in animals in Saudi Arabia

A cross-sectional study was conducted in 2018 in the eastern region of Saudi Arabia to determine the incidence of MRSA in goat farms [98]. Healthy goats and goats with respiratory symptoms such as nasal discharge and cough were included in this study [98]. MRSA was found in nasal swabs of 2% of 775 goats, and all MRSA isolates tested positive for multidrug resistance, as was 23.5% of the MSSA isolates from the same study [98]. In the same region, a veterinary clinic admitted 400 cats, of which 75 were isolated from healthy and ill cats, and 15 tested positive for MRSA [108]. The MRSA sequence types ST80 and ST239 have been isolated from healthy cats, whereas ST22, ST8, ST5, ST71, ST80, and ST239 have been isolated from infected cats [108]. The isolation of MRSA ST80, a CA-MRSA clone lineage, from cats highlights the cross-transmission of MRSA between cats and humans [108]. The incidence of MRSA among domestic animals in the Saudi Arabian region of Qassim was previously investigated by Alzohairy [99], and the antibiotic susceptibility profile was collected between January and April 2010. This showed that camels had an unexpectedly high prevalence of MRSA colonization, reaching up to 35.5%; however, the colonization rates of MRSA in sheep, goats, and cows are fairly low [99]. Notably, the highest prevalence of multidrug-resistant MRSA strains was observed in camel isolates (41.1%), whereas MRSA strains isolated from sheep, cows, and goats exhibited similar rates of multidrug resistance [99]. Table-1 [85, 86, 92, 95–97] provides a detailed distribution of MRSA detected in animals in Saudi Arabia. Hemeg *et al*. [109] reported MRSA in pets and their owners; swabs revealed a prevalence of MRSA in dogs of 44.4%, whereas cat samples revealed an MRSA prevalence of 27.3%, and the owner’s swabs detected 30 MRSA isolates with a prevalence rate of 42.8%. MDR isolates were found in 45 *S. aureus* isolates at a rate of 69.2% [109]. These findings demonstrate that companion animals such as dogs and cats are *S. aureus* reservoirs and a source of public health risks, particularly with MRD *S. aureus* [109]. *S. aureus* and MRSA were isolated from the mucus membranes and respiratory tracts of farm rats in Al-Ahsa in Eastern Saudi Arabia [110]. All *S. aureus* isolates obtained from rats were penicillin-resistant (100%), with extremely high resistance to imipenem (71%) and a last-line antibiotic [110]. The percentage of isolates resistant to other *β-*lactams is also high [110]. Some data suggest that methicillin-resistant staphylococci can be transmitted between goats and their surroundings and between goats and humans [103]. In the Eastern Province of Saudi Arabia, seven of nine methicillin-resistant staphylococci isolates found in goats and their environmental surroundings were MRSA. All MRSA isolates identified in this study belong to clones previously known to infect humans, including isolates harboring ST6, ST5-SCC*mec* VI, and ST5-SCC*mec* V [103].

**Table-1:** Distribution of methicillin-resistant *Staphylococcus aureus* detected in animals in Saudi Arabia.

Region	City	Animals in which *S. aureus* was found	MRSA detection	Sampling time	Samples	Healthy/sick	Clonal lineages of MRSA	References
Eastern	-	Goats	Yes	From Jan. to Dec. 2015	Nasal swabs	Healthy/with respiratory symptoms		[85]
Eastern	-	Cats	Yes	From Jan. to Dec. 2018	Nares, conjunctival sac, skin, and ear canal	Healthy and diseased	ST80, ST22, ST239, ST8, ST5	[95]
Central	Qassim	Camels, sheep, cows, and goats	Yes	From Jan. to April 2010	Nasal swabs	Healthy	-	[86]
-	-	Companion animals (Cat and dogs)	Yes	-	Nasal and ear swabs	Infected	-	[96]
Eastern	Al-ahsa	Rats	Yes	2018	Nasal and rectal swabs	-	-	[97]
Eastern	-	Goats and their farm environments	Yes	Nov. 2019 to Aug. 2020	Goat’s nasal swabs, goat’s milk, goat’s drinking water, and farm soil	-	ST5,ST6	[92]

### MRSA detection in food samples from Saudi Arabia

In Saudi Arabia, staphylococci have been linked to 41% of “bacterial food poisoning” cases and have been reported in processed cheeses as well as raw beef, camel, and dairy [111]. Scarce research exists regarding the occurrence of drug-resistant staphylococci in the Saudi Arabian environment and food supply [112]. MRSA may spread through food-processing industries and slaughterhouses [113]. The probable cause of MRSA contamination at slaughterhouses is the mobility of animals and workers; however, in food processing industries, the primary predisposing factors are poor hygienic conditions of employees, machinery, utensils, and the surroundings [5]. MRSA-infected or carrier personnel can spread MRSA in an unsanitary setting in butcher shops, factories, or restaurants to foods such as ready meals, raw beef, poultry, camels, and milk products [5]. Notably, public health concerns about the association of MRSA with food-borne illnesses have grown because of its access to human food [58]. Table-2 [14, 99, 103-106] provides detailed information on the detection of *S. aureus* in food from animals in Saudi Arabia. In Riyadh, Saudi Arabia, 100 samples of fresh retail meat, including camel, beef, lamb, and chicken, contained 25 *S. aureus* isolates, of which camel meat had the highest MRSA contamination rate (20%) compared with other types of meat, and a unique clone of MRSA (CC-15) was found in fresh camel meat [15]. The MRSA lineages CC1, CC88, and CC80, which were identified in retail meat, are well-recognized human-associated clonal types, implying contamination of retail meat with human MRSA strains [15]. In the same study, oxacillin-susceptible *mec*A-positive *S. aureus* (OS-MRSA) isolates were identified for the 1^st^ time in retail meat in Saudi Arabia [15]. A decade ago, *S. aureus* and coagulase-negative staphylococci (CONS) were found to be prevalent in food and the environment in Makkah, Western Saudi Arabia [111]. *S. aureus* was detected in raw milk, cheese, mucosal membranes, and, to a lesser extent, biofilm [111]. *S. aureus* and CONS revealed considerable resistance to beta-lactams and glycopeptides, and both coagulase-positive and coagulase-negative isolates from all different types of samples exhibited multidrug resistance [111]. The prevalence of *S. aureus* and MRSA in processed food samples was examined in Riyadh, where 150 processed food items were analyzed for *S. aureus*, including salami, sausages, and smoked turkey; 94 (62.6%) samples tested positive, including 53 (56.3%) isolates that were confirmed to be MRSA [11]. The majority of MRSA isolates were multidrug resistant, and vancomycin-resistant genes were found in 33.3% of MRSA *mec*A-positive isolates [11]. Another comparable study conducted in 2021 examined the incidence of MRSA in dairy products in Riyadh, Saudi Arabia, in which 70% of 100 collected dairy product samples were *S. aureus-positive*, and 72.9% of these isolates were identified as MRSA [114]. In addition to the propensity of *S. aureus* to form biofilms and resistance to biocides, improper handling, processing, and storage of food can result in contamination of food with this pathogen [115]. In addition, a high percentage of MRSA isolates were multidrug-resistant, with 28.6% of MRSA-*mec*A-positive isolates carrying vancomycin resistance genes [114]. In addition, to estimate the frequency of antibiotic resistance in various food-borne bacteria in meat, a team of researchers gathered samples from local and imported meat (beef, camel, lamb), chicken, and other foods from domestic retail stores in the Riyadh area [116]. Approximately 24% of *S. aureus* were found among the samples, indicating that *Enterococcus* spp. and *S. aureus* have broad resistance to erythromycin [116]. A study performed in 2020 also discovered that pasteurized camel milk contains a considerable amount of MRSA bacteria (10%) [117]. The use of temperatures higher than those used for pasteurization may have enabled the evolution of heat-resistant strains, resulting in the production of novel proteins that are resistant to higher temperatures. This incidence is alarming and poses a serious threat to public health [117].

**Table-2:** Detection of *Staphylococcus aureus* in food from animals in Saudi Arabia.

Region	City	Samples	The number of samples studied	Sampling time	Raw/cooked	Detection of MRSA	*S. aureus* prevalence	References
Central	Riyadh	Camel milk	100	March 2017 and May 2017	Pasteurized	Yes	Yes	[106]
Central	Riyadh	Fresh retail meat (Camel, beef, lamb, poultry)	100	March to December 2014	Raw	Yes	Yes	[14]
Western	Makkah	Food and Environmental samples (Milk, cheese, taps, and taps filter swabs)	59	December 2010	Raw milk and processed cheese	Yes	Yes	[99]
Central	Riyadh	Processed food (Smoked turkey, sausages, and salami)	150	August 2019 to March 2020	Processed	Yes	Yes	[103]
Central	Riyadh	Milk and dairy products	100	August 2019 to March 2020	Raw milk (Goat milk, horse milk, camel milk, and cow milk) and unpacked cheese	Yes	Yes	[104]
Central	Riyadh	Unprocessed meat samples (Camel, beef, lamb, poultry)	288	September 2009 to January 2010	Raw	-	Yes	[105]

## Conclusion

Some of the MRSA clones (ST80, ST22, ST239, ST8, and ST5) detected in animals have been linked to CA-MRSA clones in Saudi Arabia, suggesting that these isolates may have human origins [103, 108]. In addition, recently identified MRSA clones (*CC15, CC1153, and ST1156*) in Saudi Arabia have been reported in food and animals [15, 19, 104]. Therefore, the high prevalence of MRSA isolates found in food that share clonal lineages with MRSA isolates from animals in Saudi Arabia indicates potential MRSA transmission from animals and food to humans, and vice versa. Although the number of MRSA studies in Saudi Arabia has increased in recent years, they are confined to specific cities and are mostly concentrated on clinical *S. aureus* isolates. In addition, these investigations showed *S. aureus* in food and animals, but only a small proportion of the samples underwent molecular typing. Consequently, further investigations are required to assess the role of food produced by animals and livestock in the transmission of *S. aureus* and MRSA. Surveillance of *S. aureus* and MRSA in humans, animals, and food can help understand MRSA epidemiology and implement effective management practices.

## Authors’ Contributions

DMA and MMA: Conceptualized and designed the study, conducted a comprehensive literature search, analyzed the data, and drafted the manuscript. Both authors have read, reviewed, and approved the final manuscript.
